# Multivariate Analysis of Structural and Functional Neuroimaging Can Inform Psychiatric Differential Diagnosis

**DOI:** 10.3390/diagnostics11010019

**Published:** 2020-12-24

**Authors:** Drozdstoy Stoyanov, Sevdalina Kandilarova, Katrin Aryutova, Rositsa Paunova, Anna Todeva-Radneva, Adeliya Latypova, Ferath Kherif

**Affiliations:** 1 Department of Psychiatry and Medical Psychology and Research Institute at Medical University of Plovdiv, 4000 Plovdiv, Bulgaria; sevdalina.kandilarova@mu-plovdiv.bg (S.K.); Katrin.Aryutova@phd.mu-plovdiv.bg (K.A.); rositsa.paunova@mu-plovdiv.bg (R.P.); anna.todeva@mu-plovdiv.bg (A.T.-R.); 2 Centre for Research in Neuroscience—Department of Clinical Neurosciences, CHUV—UNIL, 1010 Lausanne, Switzerland; Adeliya.Latypova@chuv.ch (A.L.); Ferath.Kherif@chuv.ch (F.K.)

**Keywords:** multivariate linear method, validation, diagnosis, discriminative, signatures of disease, schizophrenia, depression

## Abstract

Traditional psychiatric diagnosis has been overly reliant on either self-reported measures (introspection) or clinical rating scales (interviews). This produced the so-called explanatory gap with the bio-medical disciplines, such as neuroscience, which are supposed to deliver biological explanations of disease. In that context the neuro-biological and clinical assessment in psychiatry remained discrepant and incommensurable under conventional statistical frameworks. The emerging field of translational neuroimaging attempted to bridge the explanatory gap by means of simultaneous application of clinical assessment tools and functional magnetic resonance imaging, which also turned out to be problematic when analyzed with standard statistical methods. In order to overcome this problem our group designed a novel machine learning technique, multivariate linear method (MLM) which can capture convergent data from voxel-based morphometry, functional resting state and task-related neuroimaging and the relevant clinical measures. In this paper we report results from convergent cross-validation of biological signatures of disease in a sample of patients with schizophrenia as compared to depression. Our model provides evidence that the combination of the neuroimaging and clinical data in MLM analysis can inform the differential diagnosis in terms of incremental validity.

## 1. Introduction

Schizophrenia (SCH) and depression are psychiatric disorders that have a very high prevalence in psychiatric clinical care and cause immense social burden in terms of disability and health care costs [1]. They are amongst the most detrimental and socially significant disorders which lead to chronic disability of the patients. Those individuals have an average mortality rate that is 2-3 times greater than the general population, resulting in a reduced lifetime of 10 to 20 years [2]. Both schizophrenia and depression are associated with a high risk of comorbidity with somatic illnesses as well as other psychiatric disorders, which leads to serious health consequences in addition to the substantial risk of self-inflicted death [3,4]. Therefore, it is crucial to make advances in the diagnostic and therapeutic approaches to improve the prognosis and outcome of these debilitating conditions. [5].

However, this essential enterprise is hugely intertwined with the undefined framework of psychiatric nosology and categorization. Thereupon, contemporary research has drifted towards a paradigm shift, namely the Research Domain Criteria Project (RDoC) comprehending mental disorders not as distinct entities but as spectral dimensions encompassing their biological, psychological and phenomenological features [6]. A number of studies exploring the symptomatologic overlap among different disorders have demonstrated the existence of neurobiological alterations associated with various dysfunctions which transcend beyond the categorical classification of SCH, bipolar disorder, and major depressive disorder (MDD) [7,8]. Moreover, in the affective dichotomy of BD II and MDD the subthreshold hypomanic syndrome is not reflected properly in the formulation of MDD diagnosis in clinical practice [9]. Therefore, it is difficult to assume clear and uncontestable cases of MDD, especially in the longitudinal sense, which is valid for our sample as well. In addition, there is a lack of valid consensus-based biomarkers to underpin the clinical diagnosis in psychiatry [10].

In order to address this, our research team has implemented a novel paradigm design directed towards the cross-validation between self-assessment scales and functional MRI [11,12,13]. We have used von Zerssen’s Paranoid Depression Scale as it captures symptoms spanning across the spectrum from psychosis to depression. In the initial experiment we used items from the depression subscale in patients with depression and healthy controls, which resulted in establishing the sensitivity of the method, i.e., the distinction between pathological and normal states [11]. Subsequently, we developed the experimental paradigm by including items from the paranoid subscale with the aim to determine the specificity (i.e., distinction between two pathological states) as well, by its implementation in two groups of patients—schizophrenic and depressed. While the standard statistical methods did not allow for a significant differentiation between the groups in a direct comparison [12] the use of the multivariate linear model (MLM) resulted in the identification of specific brain signatures with a high level of prediction accuracy reaching 90% [13].

However, one of the critical caveats in the interpretation of MRI data in psychiatry is multiple realizability [14]. Alterations in different regions are reported to be implicated in the pathogenesis of one and the same phenomenon in psychopathology (symptom or sign) and different clinical phenomena are often explained by changes in one and the same brain region. This may well be due to confound originating from the research design such as sample structure, criteria for exclusion and inclusion, medication, gender, and age co-variates. Furthermore, it may also be due to the biased application of one or another MRI modality (structural, functional resting state, and task related).

Recently, the multimodal neuroimaging approach plays an increasing role in elucidating the structural and functional properties of a healthy or abnormal brain. Such computational methods are also valuable for clinical research on the dynamics of disease development [15]. An illustration is multimodal fusion, where the objective is to focus on the strength of each imaging modality and its interrelationships as a compound entity instead of an independent analysis. Thus, each imaging approach represents an aspect of the function and/or structure of the brain and the data fusion translates it into a collaborative space, providing an important tool to help uncover the underlying pathobiological mechanisms of mental disorders. In addition, the method allows for a composite analysis including augmentation of the neuroimaging sequences with modalities such as genetic data [16], aiming at the possibility for computational classification of psychiatric disorders [17,18].

Although the existing methodological gaps are a significant confounding factor for the clinical application of such approaches [19], scientific research has provided substantial evidence for the potential future implications in the study, diagnosis and treatment of disorders of the central nervous system (CNS). Latest advancements in data fusion transcend the usage of conventional general linear model-based approaches attempting a convergence of several (task) fMRI data sets from the same individual in order to specify common versus specific sources of activity [18]. Furthermore, an evidence-based determination of the functional significance of certain brain regions and activation changes in brain disorders enhances the confidence and reliability of the methods. The reciprocal interpolation of functional and structural modalities may also provide more informative insight into identified alterations of brain architecture and/or connectivity patterns [20].

Investigating several data sets (e.g., combining functional Magnetic-Resonance Imaging, Diffusion Tensor Imaging and structural Magnetic-Resonance Imaging (fMRI-DTI-sMRI)) in a comparison between patient and control groups is an innovative attempt, which may be used in the study of various neuropsychiatric disorders or subsets of a particular disorder (such as psychotic or non-psychotic bipolar disorder) [21]. In addition, the utilization of machine learning algorithms as an analytical entity of fMRI data offers the opportunity not only to expand the interdisciplinary exploration of the etiopathogenesis of psychiatric disorders but also to accelerate the process of translation between science and clinical practice [22].

In this context, we conceptualized our current study along the hypothesis that multimodal imaging can allow for better definition of the fundamental biological signatures of paranoia and depression as the two-dimensional extremities of the hypothesized diagnostic spectrum via the combination of the fMRI signal in three different neuroimaging modalities—structural, resting state and task related fMRI. We used multivariate linear model (MLM) as a method, which not only permits the processing of vastly dimensional data [23], but has also been established as reliable in the application to different neuroimaging techniques [24,25]. Thus, the aim of the present study was to assess to what extent the combination of these different imaging modalities i.e., resting state and task related fMRI, along with structural MRI can contribute to the differentiation of these major psychiatric disorders.

## 2. Materials and Methods

### 2.1. Participants

The current study recruited a total of 44 patients of whom 19 with schizophrenia (mean age 39.3 ± 14.8 years, 9 males), and 25 with current depression (*n* = 25, mean age 44.2 ± 12.1 years, 9 males): Unipolar (*n* = 10, mean age 43.7 ± 13.2 years, 5 males) and bipolar 2 (*n* = 15, mean age 44.5 ± 11.8 y, 4 males). Each patient was assessed by an experienced psychiatrist (D.S, S.K., K.A.) by means of a general clinical interview and the structured Mini International Neuropsychiatric Interview (M.I.N.I 6.0) [26]. The Montgomery–Åsberg Depression Rating Scale (MADRS) [27] and the Positive and Negative Syndrome Scale (PANSS) [28] were used in addition to assess the severity of the symptoms. The cut-off for inclusion of depressed patients was set to a minimal total MADRS score of 20, while for schizophrenia and individual score on PANSS P1 (delusions) or P6 (suspiciousness) of 3 was required. All patients had a steady pharmacotherapeutic regime within 14 days before inclusion. In patients with schizophrenia, drugs from the pharmacological group of antipsychotics predominated, while in the group of depressed individuals the majority took antidepressants and/or mood stabilizers (anticonvulsants). The most commonly taken drugs in the paranoid group were atypical antipsychotics. One of the subjects in the paranoid group did not take any medication. Among the most common medications in the depression group were Selective serotonin reuptake inhibitors (SSRIs) and Serotonin–norepinephrine reuptake inhibitors (SNRIs), in combination with mood stabilizers and tranquilizers. There was one patient in the depressed group who was not taking medication.

Subjects were excluded in the following cases: Age under 18 or over 65, presence of metal implants in the body that are not compatible with MRI, comorbid mental disorders, any severe somatic or neurological disease, and history of traumatic brain injury with loss of consciousness.

### 2.2. Image Acquisition

All participants underwent a scanning procedure performed on a 3T MRI system (GE Discovery 750w). The protocol included three different MRI sequences: First a high resolution structural scan (Sag 3D T1 FSPGR), slice thickness 1 mm, matrix 256 × 256, TR (time of relaxation) 7.2 msec, TE (echo time) 2.3 msec, and flip angle 12°, followed by a resting state functional scan with eyes closed (2D EPI sequence), slice thickness 3 mm, 36 slices, matrix 64 × 64, TR 2000 msec, TE—30 msec, flip angle 90°, 192 volumes and concluding with a task sequence (see following paragraph), slice thickness 3 mm, matrix 64 × 64, TR 2000 msec, TE 30 msec, and flip angle 90°, 256 volumes. Each of the two functional scans started with 5 dummy time series which were automatically excluded from the image processing.

### 2.3. fMRI Task

E-prime software (Psychology Software Tools, Inc) was used to construct the paradigm which consisted of 32 s blocks with three different active conditions and one 20 s block with the rest condition. As it is has been extensively described in our previous work [12], a brief summary will be given in the following lines.

The stimuli were written statements from the von Zerssen’s paranoia-depression scale and from a questionnaire of general interests. There were Depression Specific (DS) blocks with the statements from the depression subscale (“I cry easily”, “I feel melancholic and depressed”), and Paranoid Specific (PS) blocks from the paranoia subscale (“Somebody wants to kill me”). The Diagnostically Neutral (DN) blocks included statements from a questionnaire about general interests and likes (such as “I like to repair household appliances” etc.). The participants were instructed to read the statements carefully and to respond with a button press according to their level of agreement. There were four possible answers (“completely true”, “mostly true”, “somewhat true”, “not true”) and respectively four response buttons (upper left, lower left, lower right, upper right) presented on the screen under each statement. The paradigm consisted of four active blocks of each type, alternating between the three conditions but always followed by the rest condition—fixation cross (DS__rest__DN__rest__PS__rest).

### 2.4. MRI Data Analysis

#### 2.4.1. Voxel-Based Morphometry

SPM 12 (Statistical Parametric Mapping, http://www.fil.ion.ucl.ac.uk/spm/) software running on MATLAB R2020 for Windows was used for the analysis of the structural MRI images. Spatial preprocessing included first individual segmentation followed by normalization to the Montreal Neurological Institute (INM) template created with a diffeomorphic anatomical recording using exponentiated lie algebra (DARTEL; Ashburner, 2007). Finally, the resulting modulated grey matter volume estimate was smoothed with a 3D Gaussian kernel (8 mm full width at half height, FWHM) to account for the individual anatomical differences. Total intracranial volume (TIV) was derived for each participant and included as a covariate in the analyses to account for global individual differences in head size.

#### 2.4.2. Task-Related Functional Data Processing

The functional images acquired during the task were realigned, co-registered with the anatomical image, normalized to MNI space, and spatially smoothed with an 8 mm FWHM Gaussian kernel. General Linear Model (GLM) was then applied to the time series, convolved with a canonical hemodynamic response function (HRF). The design matrix included the six rigid body motion correction parameters as covariates of no interest. Individual F-contrasts were defined for all active conditions orthogonal to the motion effect to be further used for the MLM analysis.

#### 2.4.3. Resting State Data Processing—Whole Brain Residual Partial Activations

The images acquired during resting state were processed in the same way as the task-related fMRI images—realignment, co-registration, normalization and smoothing. These processing steps were followed by the application of a GLM with a canonical HRF convolution to the time series. The individual residual mean square images were used for the consequent first level MLM analysis.

#### 2.4.4. MLM Analysis

To identify the brain signatures which encompass most of the differences between the diagnoses and between the different mapping modalities, we used a multivariate method, namely the Multivariate Linear Model—MLM (https://github.com/LREN-CHUV/MLM). MLM is a data driven approach which has shown great potential for summarizing and capturing the components of individual differences across multiple areas. The method has wide applicability for statistical reference, predictive approach, and statistical mapping. We extended the MLM root method to a multi-level approach in order to capture multi-scale latent variables in hierarchically organized data.

To adapt to our data sets and the corresponding assumptions, we have implemented a two-step procedure. In the first step, we performed an MLM analysis of each of the modalities with the constraints operationalized in an F-test for the differences between the two diagnostic groups. The procedure identified the optimal brain mapping signature (or eigen-image) discriminating between the two groups of diseases. The method also produced a subsystem load displaying the discriminative information but at a subject level.

**Figure 1:**
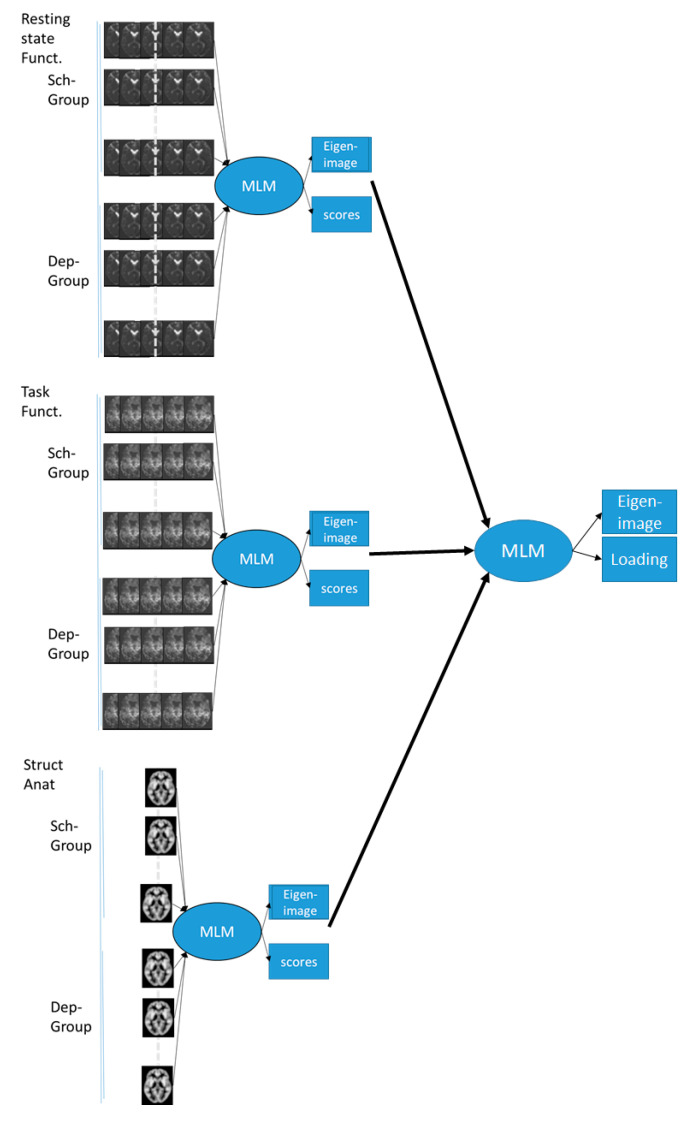
Schematic representation of the multimodal multivariate linear method (MLM) approach. Multi-modal MLM takes place in two stages. First, separate MLMs are done for each modality. Each MLM analysis will identify the eigen-image (brain signature) and the corresponding subjects’ scores that best explain the differences between the two groups. In the second step, we use MLM and the eigen-images (or the scores) from the previous stage to identify the best combinations of the modality-specific signatures and the corresponding combined brain signatures.

At the second step, we also performed an MLM analysis using the results (clean image and eigen-components) from the first step. Thus, we attempted to find the optimal combination from the previous mapping which best explained the difference between the diagnostic groups, so that theoretically we could identify up to 3 of these components (Figure 1).

### 2.5. Statistical Analysis

SPSS 22.0 for Windows was used for the statistical analysis of the demographic and clinical characteristics of the participants. Continuous variables were tested with Student’s *t*-test while categorical ones—with Chi-square test. The threshold for the level of significance was set to *p* < 0.05 for all tests.

## 3. Results

### 3.1. Demographic and Clinical Characteristics

The two patient groups were not significantly different in their demographic and clinical characteristics such as age, education level, age at onset, and illness duration. Table 1 shows the characteristics we have controlled for in the sample.

### 3.2. MLM Analysis

#### 3.2.1. Modality Specific MLM

**Figure 2:**
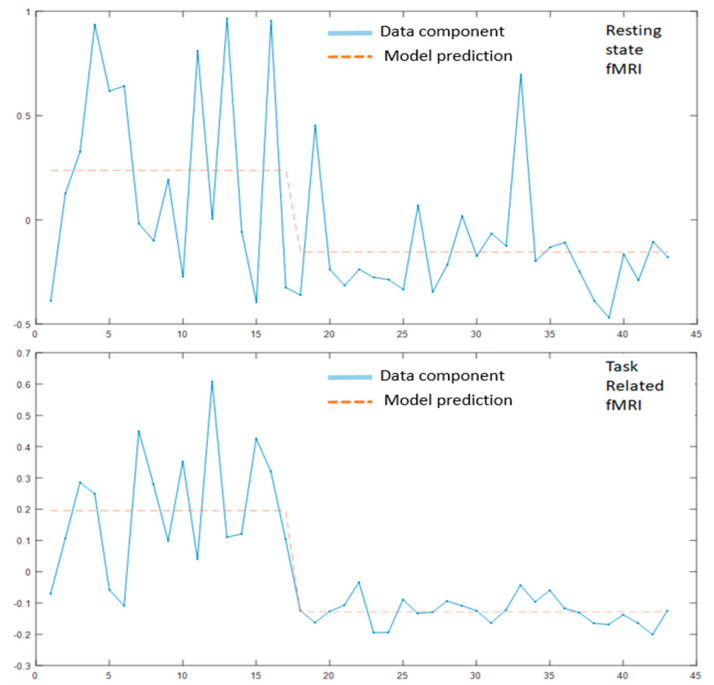

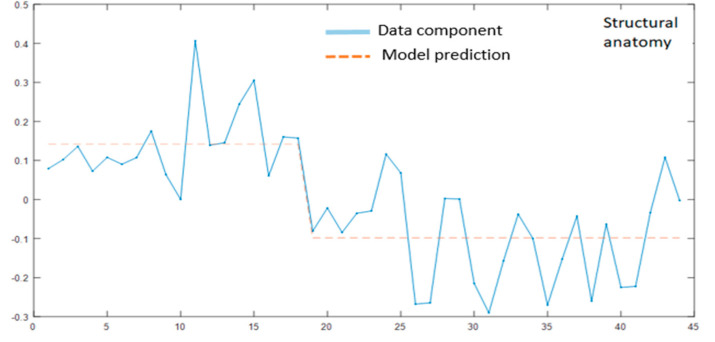
MLM components and subjects score for each of the modality. The first subjects, to the left (x-axis = N of subjects) correspond to the schizophrenia group and the rest to the depression group. The blue lines correspond to the calculated empirical components and the red dashed lines correspond to its projections/prediction in the space defined by the condition of interest, i.e., the differences between the two diagnostic groups. First segment of the figure shows the specific components identified using the resting state fMRI data, second segment shows the eigen-components which best summarize the fMRI task-related data; last segment shows the specific components for the anatomical differences between the two patient groups.

MLM was applied separately to the data from all three modalities combined with a similar model (Figure 2) that included a covariate for patient groups and adjustment covariates (age, sex, IVR).

#### 3.2.2. MLM Analyses across the Modalities

For this analysis, we used the electronic images from the first stage as input. Note that the subject loads can be used instead, and the results will remain the same (the reason is that subject spaces and the picture space are doubled, i.e., one space can be derived from another by a simple matrix transformation). Presently, we report the results found in the image spaces.

**Figure 3:**
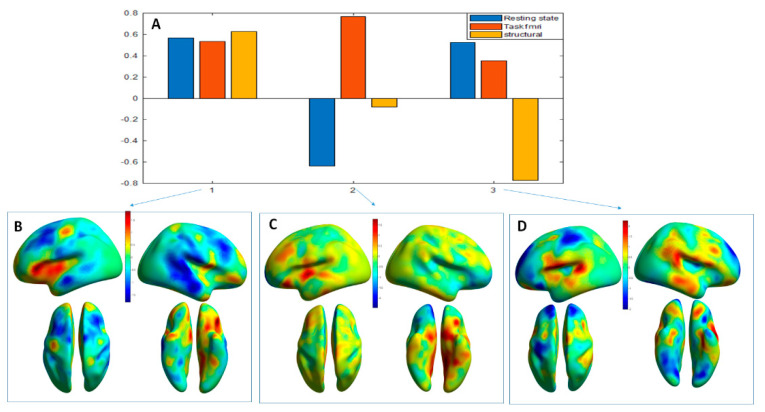
Second stage MLM analysis across modalities: The top figure (**A**) represents bar plots for the three components (1,2,3) with the contribution of each modality (resting state fMRI—blue, task fMRI—red, structural MRI—yellow). For the first component, the contribution is almost equal for all the modalities. The second bar plot, shows that that the second component is driven by the functional modalities. The last bar plot, shows that the largest contribution in the third component is from the anatomical brain signature. (**B–D**) are the corresponding eigen-images calculated by MLM projected on a 3D surface. The voxel values in the eigen-images represent the correlation of the value across all subjects at that voxel with the identified principal components.

We found that all eigenvalues were not null for the 3 components, which means they are all informative. The variance explaining the difference between the diagnostic groups by each component was respectively (35%, 33%, and 32%). Figure 3 shows the optimal contribution to each of these components.

The first component shows an equal contribution of the 3 modalities. Figure 3B shows the contribution (positive or negative) of the voxels to the mapping which corresponds to the first component.

The second component shows a difference between the idle state and the functional data related to the task. The contribution of the structural anatomy is low. Figure 3C shows the mapping corresponding to the third component, which is characterized by a larger contribution of the structural modality.

**Figure 4:**
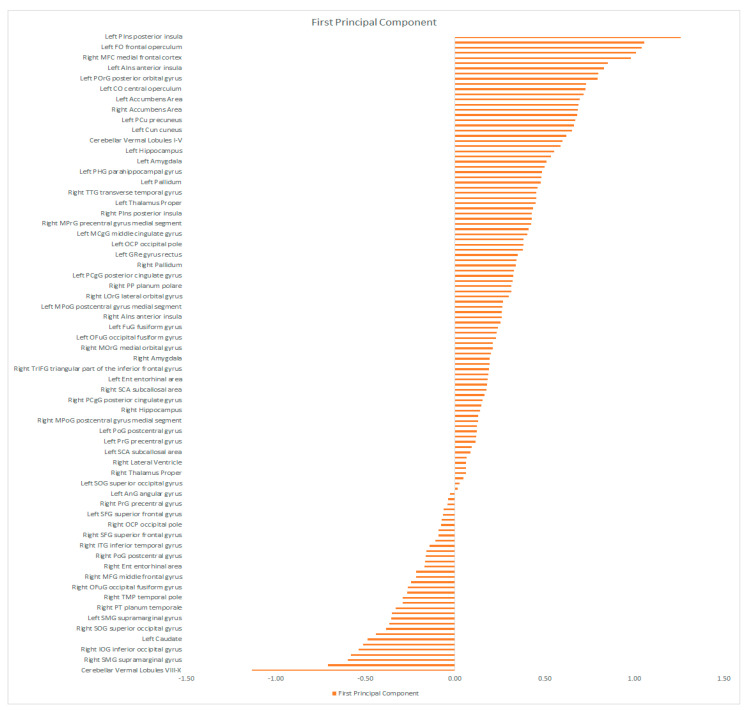
The bars plot shows the average contribution for the first component/Eigenimage computed at the regional level using the Neuromorphometric atlas. The higher the value, the higher the contribution positively or negatively.

**Figure 5:**
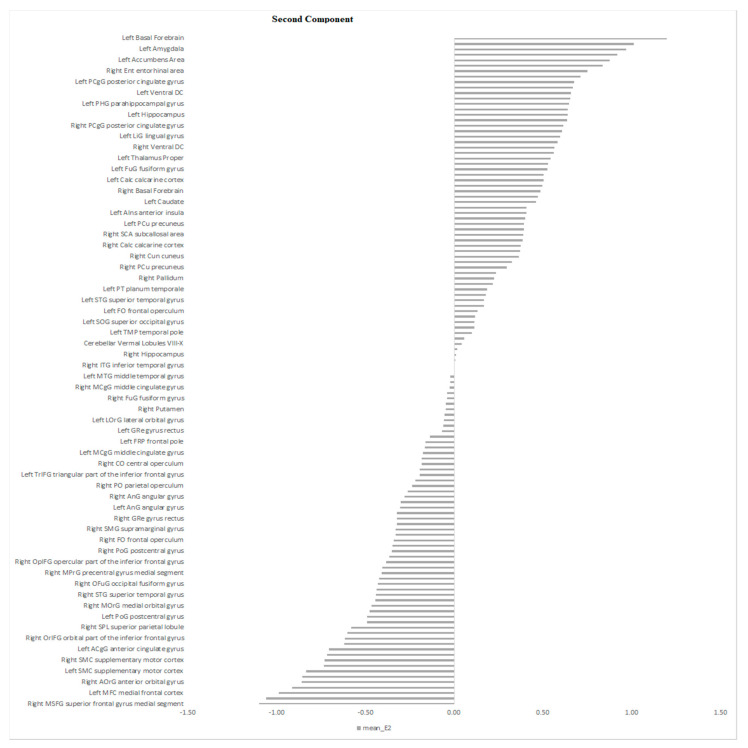
The bars plot shows the average contribution for the Secondcomponent/Eigenimage computed at the regional level using the Neuromorphometric atlas. The higher the value the higher the contribution positively or negatively.

**Figure 6:**
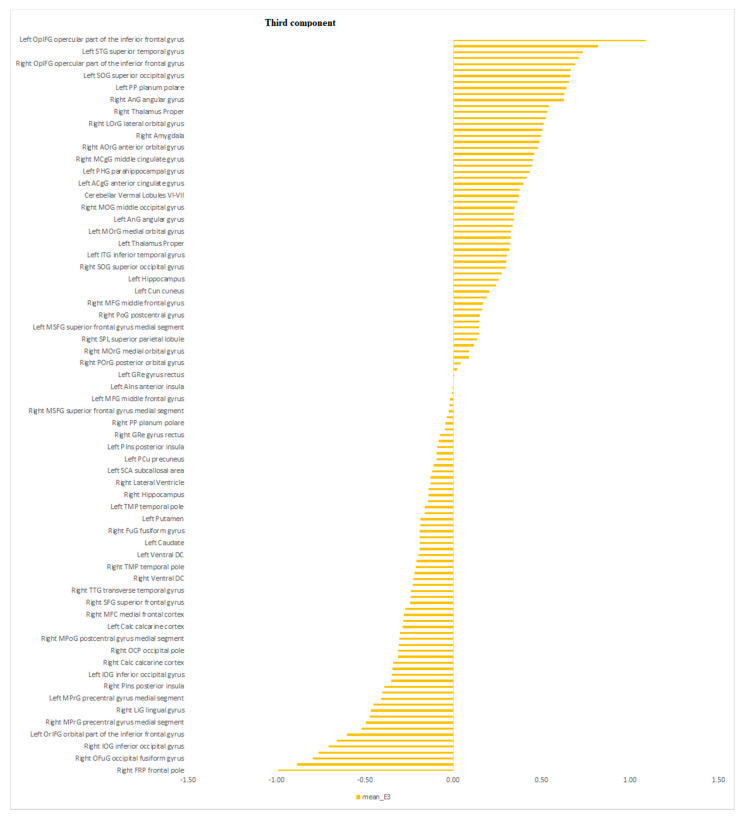
The bars plot shows the average contribution for the third component/Eigenimage computed at the regional level using the Neuromorphometric atlas. The higher the value the higher the contribution positively or negatively.

The eigen-images above were automatically parcellated into 114 gray matter regions based on Neuromorphometrics atlas (containing cortical and subcortical structures) using the SPM atlas function (spm_atlas in SPM12). To identify which regions were contributing the most to the combined brain signature across modalities, we calculated the average of the voxel’s projections from the maps in Figure 3A–D for all regions of the Neuromorphometrics atlas. The results are shown in the Figure 4, Figure 5 and Figure 6 for the first, second and third component respectively.

## 4. Discussion

The main highlight of our study demonstrates the differential contribution of the various MRI modalities as combined in principal components (PC) to brain signatures with high capacity for discrimination of the two diagnostic entities studied (schizophrenia and depression). In PC1 the three modalities have convergent cross-validation, i.e., explanatory power of structural, resting state and functional MRI which remain in one and the same direction and encompass pathways with nodes in the Default Mode Network (DMN). PC 2 is composed of divergent cross-validation of resting state and task-related functional MRI, which means that the direction of the explanatory power of the structural and functional measures is exactly the opposite. This PC includes the effort-mode network and subcortical areas. The PC3 is driven by MRI signal in the structural MRI and covers temporal and occipital areas.

In the brain signature corresponding to PC1 the regions with the highest discriminative power were localized in left sided Planum Polare (PP), transverse temporal gyrus, opercular and orbital part of the Inferior Frontal Gyrus (IFG), insular cortex (both anterior and posterior), medial frontal cortex, basal forebrain and accumbens area (both left and right). The relevance of these regions to the two diagnostic entities studied will be discussed in the following lines.

The left Planum Polare was the most prominent structure in our study that was correlated with the most discriminative value in all three brain signatures. PP is part of the Superior Temporal Gyrus (STG), which is involved in auditory processing, including language, but also has been implicated as a critical structure in social cognition. The STG has been found to be active during processing of emotional facial expressions [29]. It was also shown to be an essential structure in the pathway of the amygdala and prefrontal cortex, both of which are involved in processes of social cognition [30]. Neuroimaging studies have found that people with schizophrenia have structural abnormalities in their STG [31]. Dysfunction in the primary auditory cortex in the anterior and middle STG and the auditory association cortex in the posterior STG is assumed to play a role in causing auditory perceptual disturbances and impaired organization of thought, respectively [32]. There is convergent data that auditory hallucinations are related to a functional network of brain areas, namely auditory and language regions of the STG and Inferior Parietal Gyrus (IPG), and speech motor regions in the IFG [33,34,35].

The portion of the frontal lobe that overlies the insular cortex is the opercular part of the inferior frontal gyrus [36]. The inferior frontal gyrus/anterior insula (IFG/AI) region is involved in complex attention and working memory processing. Ventrolateral corticolimbic control pathways, including IFG/AI, and mediodorsal corticolimbic control pathways, along with dorsal Anterior Cingulate Cortex (ACC) regions, perform partially separable but interconnected roles in adaptive behavior under environmental circumstances that vary in the degree of predictability [37]. The IFG/AI is one of the regions that activate when exhibiting anxiety and stress induced behavior [38]. In addition, antidepressant effects and sleep deprivation were associated with an activity change from IFG/AI to dorsolateral prefrontal cortex [39].

Nucleus accumbens (NAcc) which is also prominent in PC 1 is engaged in the control of emotions and affects integration. This region is a central output for dopaminergic (DA-ergic) projections and also receives glutamatergic input from the hippocampus and the prefrontal cortex [40]. The NAcc and the medial prefrontal cortex receive projections from the ventral tegmental area, which is also a DA-ergic nucleus.

The DMN is suggested to have a potential role in the integration of cholinergic and DA-ergic networks related to memory and emotions [40]. Stimulants of the Central Nervous System (CNS) like amphetamines are known to significantly increase the extracellular level of dopamine (DA) and noradrenaline (NA) in functional connectivity networks [41] via DA-ergic and noradrenergic (NA-ergic) terminals which are highly distributed in cortical areas. Administration of dextro-amphetamine (dAMPH) increases both DA and NA in the prefrontal cortex, but only DA in the striatum. As such, the regulation of connectivity networks in the striatum can be determined primarily by the release of DA, whereas the cortical functional connectivity is both affected by changes of DA and NA. The inverted U-hypothesis of DA-ergic modulation, suggesting that there is an optimal level of DA-ergic stimulation, with both too little and too much DA negatively impacting behavior, supports this finding [42]. DA strengthens the connection between the Frontoparietal Control Network (FPCN) and the DMN in the resting state where internal cognition dominates, thus reducing the relation between the FPCN and the Dorsal Focus Network [40]. These connections reveal the important role of network interaction in the modulation of attention [43].

The regions of the second brain signature that demonstrated the highest contribution to the discriminative power of PC2 were localized in the left PP, bilateral Supplementary Motor Cortex (SMC), bilateral MFC, left anterior and left posterior lingulate gyrus and right Frontal Pole (FP) along with subcortical structures such as bilateral amygdalae, left hippocampus and left parahippocampal gyrus. The modalities that contributed to PC2 were the task-related and the resting state functional MRI. The regions of its brain signature are nodes implicated in the effort-mode network and subcortical areas [44].

Effort-mode network/extrinsic mode network (EMN) is complementary to the DMN in such a way that the EMN is down-regulated during task absence times, while the DMN is up-regulated [45]. The EMN has basically a fronto–temporo–parietal spatial distribution, including the inferior and middle frontal gyri, the inferior parietal lobule, the supplementary motor area, the inferior temporal gyrus. Network up- and down-regulation dynamics dysfunction has been proposed to have neuronal implications for cognitive disability found in many psychiatric disorders such as schizophrenia [45]. Since the DMN has been defined as a mode of intrinsic neuronal activity [46], the EMN is a central network for extrinsic neuronal activity [45].

The DMN exhibits activations in the medial and posterior regions, while EMN shows activations in the lateral and anterior regions, but also in the frontal and parietal areas [47,48,49]. It is hypothesized that aberrant DMN activation could be a characteristic feature for hallucinatory experiences [50]. Auditory hallucinations can be linked to abnormally elevated resting state activity in the auditory cortex itself, irregular modulation of the auditory cortex by anterior cortical midline structures as part of the DMN, and neural miscommunication between auditory cortical resting state shifts and stimulus triggered activity [51]. Cognitive dysfunction and hypo-activation observed in patients with schizophrenia, for example, when introduced to complicated cognitive tasks may be due to inadequate interactive regulation of the DMN and EMN networks, rather than a deficit with respect to a particular brain region [45]. Glutamate (Glu), Gama-amino-butyric-acid (GABA) and other metabolites (Lactate, Aspartate, Glucose, etc.) play an important role in mediating the activity of the brain during both stimulus-induced and “intrinsic activity” [44].

The amygdala is also a significant zone in PC2. FMRI show anomalies within the corticolimbic network, including the prefrontal cortex and ACC, insula, amygdala, hippocampus and striatum [52]. All of this presents the dynamic, region- and circuit-specific stress effects that could be significant for the disturbed connectivity recorded in depressed patients [53]. There is a suggestion that persistent stress leads to hyperdopaminergic activity in the mesolimbic system which presents as social decline and suicidal behavior. In several studies it is demonstrated that hyper-responsiveness of the amygdala and related emotional regions of the brain is observed in people with schizophrenia [54] and individuals at ultra-high risk (UHR) for psychosis [55], as well as in healthy people with subclinical psychotic experiences. Amygdala hyper-responsiveness has been shown to inhibit GABAergic inter-neuron function in the hippocampus by direct projections, leading to the disinhibition of pyramidal cells and, ultimately, to increased hippocampal activity [56]. In exchange, increased transmission from the hippocampus to the striatum was found to facilitate the dysregulation of striatal dopamine which is typical in schizophrenia [57]. In studies with ketamine administration to healthy controls GABA inhibition is observed, reporting that ketamine decreases DMN connectivity and reduces reactivity of amygdala-hippocampal circuity in response to emotional stimuli [58]. Other studies found increased Glu concentrations across several corticolimbic areas in schizophrenic individuals [59].

The third component identified in our study (PC3) had opposite loads of the structural and functional (both rest and task-related) modalities and was reflected in a brain signature that involved regions localized in the left and right opercular part of the IFG, right supramarginal gyrus, left superior temporal gyrus, left anterior orbital gyrus, supplementary motor cortex, and several occipital regions. Diffusion MRI and probabilistic tractography have recently been used to demonstrate that there is greater tempo-parietal-insula connectivity in the right as opposed to the left hemisphere [60]. Another tractography research recorded that subcomponent III of the Superior Longitudinal Fasciculus, an association fiber pathway that potentially interconnects the frontal with the parietal regions of the Ventral Attention Network, is greater in the right compared to the left hemisphere [61]. These results provide an anatomical framework for the right-lateralized ventral attention network involved in the salience detection. However, the implications for the functional brain network remain unclear.

In summary resting state residual activations are detected mostly in the frontal segments of the DMN, which are predominantly dopaminergic [42], task related activations yield mainly Glutamate/GABAergic subcortical network of hippocampus and amygdala, which is consistent with other studies in the field [62], and structural alterations affect the temporoparietal network [63]. In future studies, it will be necessary to examine these correlations in more depth, as many of the patients we included in the sample had been on stable antipsychotic drug therapy in previous weeks. The majority of antipsychotic medications modulate the dopaminergic neurotransmission, so at the moment our results in terms of central dopaminergic activity can be used to form hypotheses to be tested in future projects. However, we do not fail to note the fact that our findings are consistent with the data obtained so far in the field of translational fMRI neuroscience.

## 5. Conclusions

The present study was able to demonstrate that by means of MLM applied to multimodal data sets including structural, task-related and resting state functional MRI of patients with schizophrenia and depression meaningful brain signatures with high discriminative value can be identified. The first signature reflected equal loadings of the three imaging modalities which means that the regions included (PP, IFG, Insula, NAcc etc.) have both structural and functional characteristics that can discriminate between the two groups. The second signature encompassed regions that have high discriminative power in the functional modalities i.e., task-related vs resting state fMRI and those regions are part of the EMN and DMN, respectively. The third brain signature reflected opposite loadings of the structural and functional imaging modalities and it is comprised mainly of temporo-occipital and motor regions.

The limitations of our research are related to the heterogeneity of the study population in terms of the two depression subgroups (unipolar and bipolar) and the novel design of our paradigm, which contributed to difficulties in attempting to compare the results with correlated research. An additional possible confound is the medication status of the patients. Our intention is to explore the influence of medication on the brain signatures in another study to follow. Such shortcomings may be overcome by expanding translational neuroimaging studies through separate centers using a similar approach to detecting the functional MRI substrate corresponding to the clinical self-assessment instruments in replicative protocols implemented as well in unmedicated subjects.

## Figures and Tables

**Table 1:** Demographic and clinical characteristics of the participants.

Characteristics	Schizophrenia Patients (*n* = 19)	Depressed Patients (*n* = 25)	Statistical Significance
Age (mean ± SD)	39.3 ± 14.8	44.2 ± 12.1	0.231 ^a^
Sex (M/F)	9/10	9/16	0.542 ^b^
Education (years)	13.5 ± 2.8	14.1 ± 3.5	0.548 ^a^
Age at onset (years)	27.1 ± 9.1	33.8 ± 12.4	0.139 ^a^
Illness duration (months)	142.8 ± 121.6	121.8 ± 84.5	0.505 ^a^
Episode duration (weeks)	15.4 ± 14.1	11.9 ± 10.4	0.403 ^a^

SD—Standard Deviation, ^a^ Independent samples *t*-test, ^b^ χ^2^—test.

## Data Availability

Data is available upon request.
